# Prognostic Value of Pretreatment [18F]FDG PET/CT Metabolic Parameters for Overall Survival in Esophageal Cancer: A Single-Center Retrospective Cohort with Long-Term Follow-Up

**DOI:** 10.3390/diagnostics16172757

**Published:** 2026-08-28

**Authors:** Halim Ozcevik, Muge Oner Tamam

**Affiliations:** 1 Ministry of Health, 06800 Ankara, Türkiye; 2 Department of Nuclear Medicine, Prof. Dr. Cemil Tascioglu City Hospital, University of Health Sciences, 34384 Istanbul, Türkiye; muge.onertamam@sbu.edu.tr

**Keywords:** esophageal cancer, [^18^F]FDG PET/CT, metabolic tumor volume, total lesion glycolysis, overall survival, prognosis

## Abstract

**Objective:** This study evaluates the prognostic significance of pretreatment [^18^F]FDG PET/CT metabolic parameters in esophageal cancer and identifies independent predictors of overall survival (OS). **Methods:** This retrospective single-center study included 152 treatment-naïve patients with histopathologically confirmed esophageal cancer who underwent staging [^18^F]FDG PET/CT between May 2015 and August 2025. SUVmax, SUVmean, metabolic tumor volume (MTV), and total lesion glycolysis (TLG) were measured for the primary tumor. Overall survival was analyzed with Kaplan–Meier and Cox proportional hazards models, and cut-off values were derived by receiver operating characteristic (ROC) analysis. **Results:** During a median follow-up of 70 months, 106 patients (69.7%) died. In comparisons of median values, MTV and TLG were higher in patients who died (*p* = 0.004 and *p* = 0.035), whereas SUVmax and SUVmean did not differ; both volumetric indices were associated with shorter OS as continuous variables and when dichotomized at their ROC-derived cut-offs. Their discrimination was comparable and modest (AUC 0.647 and 0.608). A multivariable model incorporating high TLG achieved a Harrell’s C of 0.715, at least as high as the primary MTV-based model (C = 0.687), so the data do not establish MTV as superior to TLG. In the primary multivariable model, distant metastasis (HR 2.46, 95% CI 1.53–3.98, *p* < 0.001), and high MTV (≥11.44 cm^3^; HR 1.79, 95% CI 1.18–2.71, *p* = 0.006) remained independent predictors of death. **Conclusions:** Volumetric [^18^F]FDG PET/CT indices (MTV and TLG) rather than SUVmax carried independent prognostic information for overall survival, alongside distant metastasis, supporting their use as an adjunct to established staging in pretreatment risk stratification, pending prospective multicenter validation.

## 1. Introduction

Esophageal cancer remains one of the most lethal malignancies worldwide, ranking among the leading causes of cancer-related death and carrying a five-year survival rate that remains below 20% in most populations [1]. Its two principal histological subtypes, squamous cell carcinoma (SCC) and adenocarcinoma, differ markedly in etiology, geographical distribution, and biological behavior: SCC predominates across East Asia and much of the developing world, whereas adenocarcinoma has risen sharply in Western and transitional settings [1,2]. Because the outcome is largely governed by disease stage and tumor biology at presentation, reliable pretreatment risk assessment is central to individualized management and to the choice among definitive (chemo)radiotherapy, neoadjuvant therapy followed by surgery, and palliative care [1,3].

Accurate pretreatment staging is central to treatment selection and outcome prediction, and the American Joint Committee on Cancer (AJCC) eighth edition tumor–node–metastasis (TNM) classification remains the cornerstone of this assessment [3]. Nevertheless, substantial survival heterogeneity persists among patients at the same anatomical stage, indicating that TNM descriptors alone do not fully capture the disease’s biological aggressiveness [3]. This limitation has driven interest in complementary staging modalities, including contrast-enhanced computed tomography, endoscopic ultrasound, magnetic resonance imaging, and positron emission tomography, each of which contributes differently to the delineation of local extent and nodal involvement [4,5,6].

Among these, [^18^F]fluorodeoxyglucose positron emission tomography/computed tomography ([^18^F]FDG PET/CT) uniquely combines anatomical localization with a quantitative readout of tumor glucose metabolism, providing prognostic information that extends beyond anatomical staging alone [6,7]. The maximum standardized uptake value (SUVmax) is the most widely reported metabolic index; however, because it is derived from a single voxel, it reflects peak metabolic intensity at one point rather than the size or extent of the metabolically active tumor [8,9]. Volumetric parameters, metabolic tumor volume (MTV), and total lesion glycolysis (TLG) integrate uptake across the whole lesion and therefore provide a more biologically representative measure of the metabolic burden of the primary tumor [8,9,10].

A growing body of evidence supports the prognostic value of these volumetric indices across the spectrum of esophageal cancer management, including definitive chemoradiotherapy, trimodality therapy, and the management of unresectable or metastatic disease [11,12,13,14]. Individual series have identified MTV and TLG as independent predictors of survival [15,16,17], sequential PET/CT studies suggest that intratreatment changes in these parameters carry additional prognostic weight [18,19,20], and systematic reviews and meta-analyses have consistently associated elevated baseline MTV and TLG with poorer overall survival [21,22]. Texture-based heterogeneity metrics have likewise been explored [23]. Interpretation across studies is nonetheless constrained by heterogeneity in tumor-delineation methodology and cut-off derivation [8], and much of the available literature is drawn from predominantly East Asian, squamous cell cohorts with relatively short follow-up, limiting generalizability to more histologically mixed populations.

Against this background, we retrospectively evaluated a contemporary single-center cohort of treatment-naïve patients with esophageal cancer, comprising both major histological subtypes, with standardized PET acquisition and long-term follow-up (median 70 months). Building on our group’s previous PET-based prognostic work in esophageal carcinoma [24], we examined baseline metabolic and volumetric parameters alongside clinicopathological variables.

We hypothesized that in a histologically mixed, treatment-naïve population of esophageal cancer patients, volumetric [^18^F]FDG PET/CT parameters, particularly metabolic tumor volume (MTV) provide prognostic information for overall survival that is independent of AJCC stage and superior to intensity-based indices such as SUVmax.

Accordingly, the primary aim of this study was to evaluate the prognostic significance of baseline metabolic and volumetric PET/CT parameters, alongside clinicopathological variables, for overall survival in a contemporary single-center cohort comprising both squamous-cell carcinoma and adenocarcinoma, with standardized PET acquisition and long-term follow-up. The secondary aims were to determine the optimal prognostic cut-off values for these parameters, to assess the association between PET-derived nodal and distant metastatic disease and overall survival, and to identify independent predictors of survival by multivariable analysis.

## 2. Materials and Methods

### 2.1. Study Design and Population

This single-center retrospective cohort study reviewed patients who underwent staging [^18^F]FDG PET/CT for esophageal cancer at the Department of Nuclear Medicine, Prof. Dr. Cemil Taşçıoğlu City Hospital. Inclusion criteria were histopathologically confirmed esophageal cancer; staging [^18^F]FDG PET/CT of adequate quality; no surgery, chemotherapy, or radiotherapy prior to PET/CT; accessible follow-up and survival data; and age ≥ 18 years. Exclusion criteria were unconfirmed histopathology, inadequate image quality, prior treatment of the primary tumor, absent follow-up data, and an active second primary malignancy. The analytic cohort comprised 152 patients. Of 185 patients who underwent staging [^18^F]FDG PET/CT for esophageal cancer during the study period, 33 did not meet the inclusion criteria (unconfirmed histopathological diagnosis, inadequate PET/CT image quality, oncological treatment prior to PET/CT, absent follow-up or survival data, or an active second primary malignancy) and were excluded; the patient flow diagram is provided as Supplementary Figure S1.

Data on esophageal cancer patients admitted to the PET/CT unit between May 2015 and August 2025 were obtained from patient files, the hospital information management system, and the Picture Archiving and Communication System (PACS). Treatment data were obtained from patient files and an electronic treatment archive system. Collected variables included patient demographics, tumor characteristics (location and histology), PET-derived nodal and distant metastatic status, and documented oncological treatment. Primary-tumor T category was not uniformly retrievable, so anatomical staging in this analysis relied on AJCC stage group and PET-derived nodal and metastatic descriptors rather than the full TNM triad. AJCC 8th edition stage group was the clinically assigned stage documented in the patient’s chart at initial diagnostic work-up, not reconstructed post hoc; because stage group is itself determined largely by nodal and metastatic status, near-complete agreement with the PET-derived nodal/metastatic descriptors used here was expected rather than an independent confirmation, and the claim that MTV adds information beyond anatomical staging should be read with this dependency in mind. For each patient, the primary-tumor metabolic parameters SUVmax, SUVmean, metabolic tumor volume (MTV), and total lesion glycolysis (TLG) were recorded, with TLG defined as MTV × SUVmean. The primary endpoint, overall survival (OS), was defined as the number of months from the date of histopathological diagnosis to death from any cause, as recorded in the Death Notification System (DNS); for surviving patients, OS was censored at the date of last follow-up. Median follow-up (70.0 months) was estimated by the reverse Kaplan–Meier method. Secondary endpoints were the association between PET-derived nodal and metastatic disease and OS, and the identification of independent prognostic factors.

### 2.2. [^18^F]FDG PET/CT Imaging Protocol

Imaging was performed after a minimum 6 h fast, provided the patient’s blood glucose concentration was ≤150 mg/dL. [^18^F]FDG was then administered intravenously at 0.09–0.14 mCi/kg (3.33–5.18 MBq/kg). After injection, patients rested in a quiet room for approximately 60 min before whole-body PET/CT acquisition. Studies were acquired on a GE Discovery MI 3-Ring system (GE Healthcare, Milwaukee, WI, USA) or a Siemens Biograph 6 LSO HI-REZ scanner (Siemens Medical Solutions, Chicago, IL, USA), employing LYSO and LSO crystal detectors, respectively. Low-dose CT was used for attenuation correction and anatomical localization, with acquisition parameters of 40–60 mAs, 140 kV, and 5 mm slice thickness. Of the 152 examinations, 78 were performed on the GE Discovery MI and 74 on the Siemens Biograph 6 system, using the manufacturers’ standard iterative reconstruction protocols. Both systems employed manufacturer-standard ordered-subset expectation-maximization (OSEM) iterative reconstruction with CT-based attenuation correction; because acquisitions spanned a ten-year period, the exact reconstruction settings (numbers of iterations, numbers of subsets, and post-reconstruction Gaussian filtering) were not uniformly retrievable for every examination. The two systems were used in parallel over the study period without a formal cross-calibration or harmonization procedure; the potential impact of scanner-related variability on quantitative parameters is addressed in the limitations. The per-scanner examination counts reported above (78 GE, 74 Siemens) were obtained by querying total examination volume logged against each scanner’s device identifier in the PACS system for the study period, rather than from a per-patient scanner field in the study database; consequently, individual patients cannot be linked back to a specific scanner in the current dataset, and a scanner-stratified sensitivity analysis of the PET-derived parameters could not be performed. Should patient-level scanner assignment become recoverable from the archive, we would be glad to provide this analysis at a later stage.

### 2.3. Image Analysis

**Figure 1:**
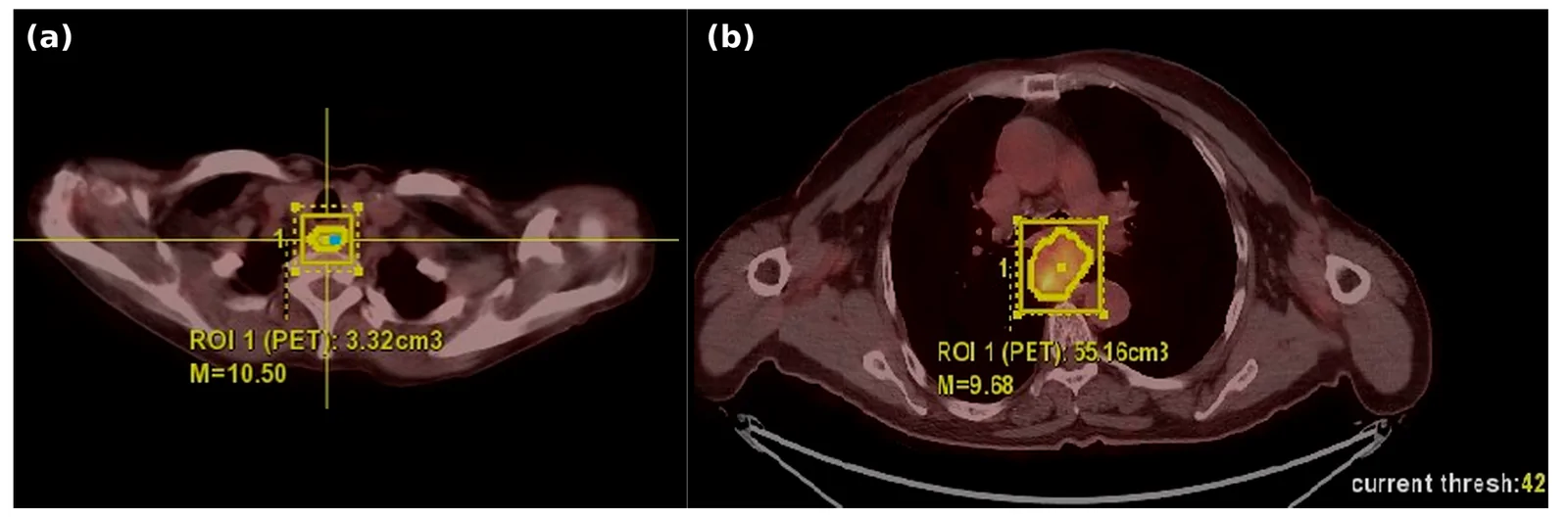
Representative pretreatment [^18^F]FDG PET/CT images illustrating primary-tumor volume-of-interest (VOI) delineation with the 42% SUVmax fixed-threshold segmentation method. Fused axial images are shown for (**a**) a patient with a low metabolic tumor volume (segmented VOI 3.32 cm^3^; SUVmax 10.50) and (**b**) a patient with a high metabolic tumor volume (segmented VOI 55.16 cm^3^; SUVmax 9.68), with the VOI outlined in yellow; the on-image label “M” denotes the workstation-reported SUVmax, and the 42%-of-SUVmax threshold used for segmentation is displayed at the lower right of panel (**b**). SUVmax is nearly identical in the two cases and well below the ROC-derived cut-off of 16.85, yet the segmented volumes differ approximately seventeen-fold and lie on opposite sides of the MTV cut-off of 11.44 cm^3^, illustrating that MTV captures tumor burden independently of peak intensity.

Images were reviewed on a dedicated workstation (GE Advanced Workstation, version 3.2; GE Healthcare). The primary esophageal tumor was first localized on maximum-intensity-projection (MIP) images and then evaluated on the fused axial, sagittal, and coronal planes. Two experienced nuclear medicine physicians independently assessed each study, and any discrepancies were resolved by consensus. Both readers reviewed all studies independently for lesion identification; quantitative measurements were obtained from a single consensus VOI after joint review, and manual adjustment of the semiautomatic VOI was limited to excluding adjacent physiological or inflammatory uptake. Interobserver reproducibility of the quantitative measurements was not formally assessed. For volumetric analysis, an ellipsoid volume of interest (VOI) was placed semiautomatically around the entire primary tumor on fused PET/CT images; its margins were then manually adjusted in three orthogonal planes to encompass the whole metabolically active tumor while excluding physiological uptake (including myocardial, gastric, and adjacent bowel activity), non-tumoral inflammatory uptake, and unrelated hypermetabolic foci, with CT morphology used to confirm the anatomical extent of the lesion. SUVmax and SUVmean were extracted from the final VOI. MTV was defined as the volume of all voxels with SUV exceeding 42% of SUVmax, a fixed relative-threshold segmentation approach widely applied in esophageal cancer PET studies [8], and TLG was computed as the product of MTV and SUVmean. Representative examples of this VOI-delineation and segmentation process are shown in Figure 1.

### 2.4. Statistical Analysis

Statistical analyses were performed using jamovi (The jamovi Project, Sydney, Australia; version 2.6.19.0). The normality of continuous variables was assessed using the Shapiro–Wilk test. Depending on their distribution, continuous data are presented as mean ± standard deviation or median (interquartile range), and categorical variables as counts and percentages. Continuous variables were compared between groups using the independent-samples t-test or the Mann–Whitney U test, and categorical variables using the Pearson chi-square test or Fisher’s exact test. Overall survival was analyzed by the Kaplan–Meier method, and groups were compared with the log-rank test. The diagnostic performance of PET/CT metabolic parameters in predicting mortality was evaluated using ROC analysis, with optimal cut-off values determined by the Youden index. Prognostic factors affecting survival were examined by univariable and multivariable Cox proportional hazards regression. The multivariable model was prespecified to include age, sex, PET-derived nodal status, PET-derived distant metastasis, and MTV dichotomized at its ROC-derived cut-off; TLG was evaluated in a separate multivariable model with the same clinical covariates, owing to its mathematical dependence on MTV and SUVmean, and AJCC stage group was examined in a separate complementary model in place of the nodal and metastatic descriptors. Treatment was not entered as a covariate because it was allocated according to a uniform institutional protocol and was itself partly determined by baseline disease characteristics (Section 2.5). Internal validation of the multivariable model, including re-derivation of the MTV cut-off in each resample, was performed by bootstrap resampling (1000 replicates) to obtain an optimism-corrected Harrell’s C statistic. Because conventional ROC analysis does not account for censoring or differences in follow-up duration, PET parameters were also retained as continuous variables in the Cox models, and time-dependent ROC analysis with inverse-probability-of-censoring weighting was performed at 12, 24, and 60 months; all data-derived cut-offs are regarded as exploratory. All quantitative PET values were verified against the original PET/CT reports and the workstation export before analysis; SUVmax, SUVmean, MTV, and TLG were available for all 152 patients, and no values were missing or imputed. Multicollinearity between AJCC stage group and the nodal and metastatic descriptors was assessed formally, and the incremental value of MTV over stage was tested by likelihood-ratio comparison of nested models. The proportional hazards assumption was assessed with scaled Schoenfeld residuals. Results were reported as hazard ratios (HR) with 95% confidence intervals (95% CI). Statistical significance was set at *p* < 0.05.

### 2.5. Treatment Protocol

All patients were managed according to a standardized institutional protocol. Neoadjuvant or definitive concurrent chemoradiotherapy (CRT) was planned for all patients, and surgery was performed on eligible patients after neoadjuvant therapy. Concurrent chemoradiotherapy consisted of weekly platinum- and taxane-based chemotherapy delivered with radiotherapy, the prescribed dose and fractionation of which depended on tumor location. In the fourth week after neoadjuvant CRT, computed tomography and endoscopic examinations were performed; the multidisciplinary team evaluated treatment response, and the decision regarding surgery was made accordingly. Adjuvant chemotherapy was administered to patients with residual tumor and/or pathological lymph-node positivity at post-surgical evaluation. Post-treatment follow-up was performed every 3–4 months for the first two years and every 6 months thereafter for up to 5 years. The specific treatment ultimately received by each patient—surgery following neoadjuvant therapy, definitive chemoradiotherapy, or palliative treatment—could not be reliably retrieved from the archival records for the full cohort and is therefore not summarized at the individual-patient level.

### 2.6. Ethical Approval

Ethical approval for this study was granted by the Scientific Research Ethics Committee of İstanbul Prof. Dr. Cemil Taşçıoğlu City Hospital (decision no. 279, meeting dated 15 June 2026). The research was conducted in accordance with the Declaration of Helsinki (1964, as revised in 2013) and applicable good clinical practice principles. Given the retrospective design and the use of anonymized archival data, the committee waived the requirement for written informed consent.

## 3. Results

### 3.1. Patient and Tumor Characteristics

The cohort comprised 152 patients (80 men, 52.6%; 72 women, 47.4%) with a median age of 63.0 years (range 21–89). Tumors were most often located in the lower esophagus (76, 50.0%), followed by the middle (55, 36.2%) and upper (21, 13.8%) thirds. On PET, 87 patients (57.2%) were node-positive and 25 (16.4%) had distant metastatic disease at staging. Pretreatment primary-tumor metabolism spanned a wide range (median SUVmax 13.99, MTV 12.83 cm^3^). Baseline characteristics are summarized in Table 1.

Histologically, squamous-cell carcinoma (SCC) was slightly more frequent (86/152, 56.6%), with adenocarcinoma comprising the remainder (66/152, 43.4%). By AJCC 8th edition stage group, most patients presented with locally advanced or metastatic disease: 62 (40.8%) were stage II, 65 (42.8%) stage III, and 25 (16.4%) stage IV. Pretreatment metabolic parameters did not differ significantly between histological subtypes: median SUVmax was 13.99 in SCC versus 13.95 in adenocarcinoma (*p* = 0.57), median MTV 12.11 versus 14.67 cm^3^ (*p* = 0.15), median SUVmean 8.79 versus 8.36 (*p* = 0.43), and median TLG 93.88 versus 114.54 g (*p* = 0.70).

### 3.2. Overall Survival

**Figure 2:**
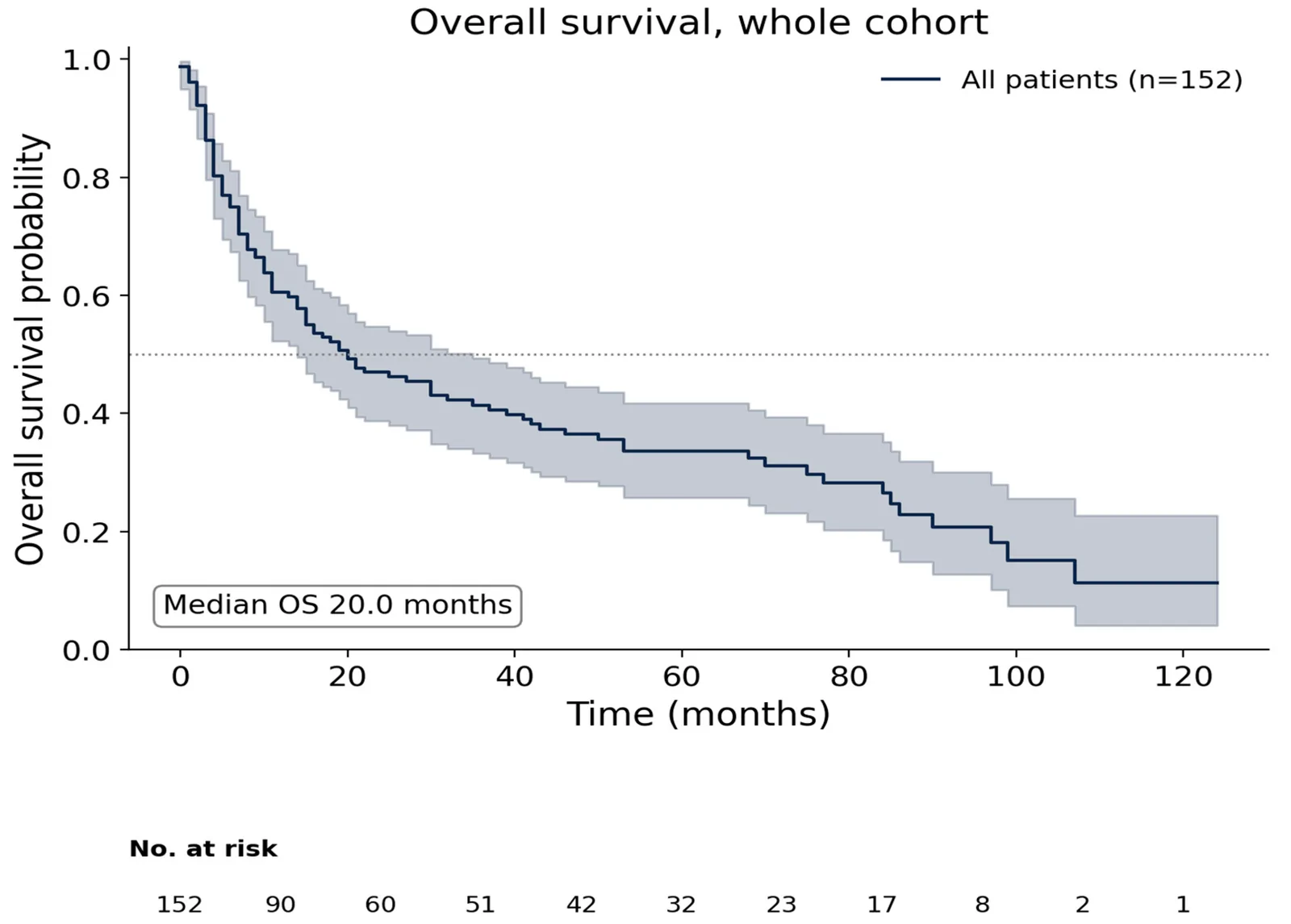
Kaplan–Meier overall survival for the whole cohort with 95% confidence interval (shaded). Numbers at risk are shown below the *x*-axis.

Over a median follow-up of 70.0 months (range 1–124), 106 of 152 patients (69.7%) died. Median OS was 20.0 months. Estimated OS was 75.0% at 6 months, 60.5% at 1 year, 47.0% at 2 years, 41.4% at 3 years, 33.7% at 5 years, 26.5% at 7 years, and 11.4% at 10 years (Figure 2).

### 3.3. Clinical Variables and Survival

**Figure 3:**
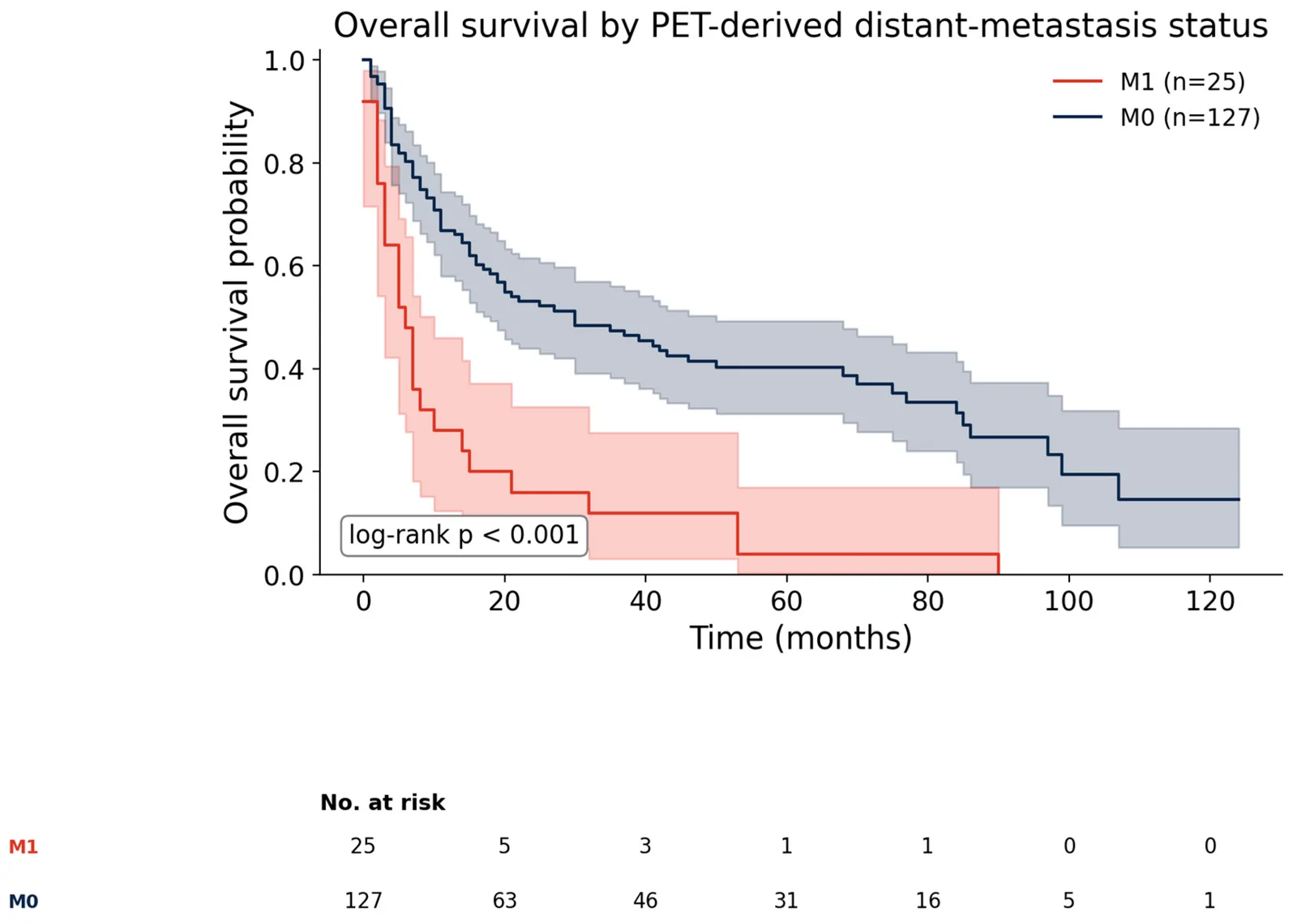
Overall survival by PET-derived distant-metastasis status (M0 vs. M1). Numbers at risk are shown below the *x*-axis.

On log-rank testing, OS differed significantly by nodal status (median 53.0 vs. 14.0 months for N0 vs. N+, *p* < 0.001) and distant metastasis (median 30.0 vs. 6.0 months for M0 vs. M1, *p* < 0.001; Figure 3). Male patients had shorter OS than female patients (median 14.0 vs. 41.0 months, *p* = 0.007), whereas tumor location was not associated with survival (*p* = 0.393). Overall survival also differed markedly by AJCC stage: median OS was 68.0 months for stage II, 16.0 months for stage III, and 6.0 months for stage IV (log-rank *p* < 0.001 for stage II vs. III–IV).

### 3.4. Metabolic Parameters and Survival

**Figure 4:**
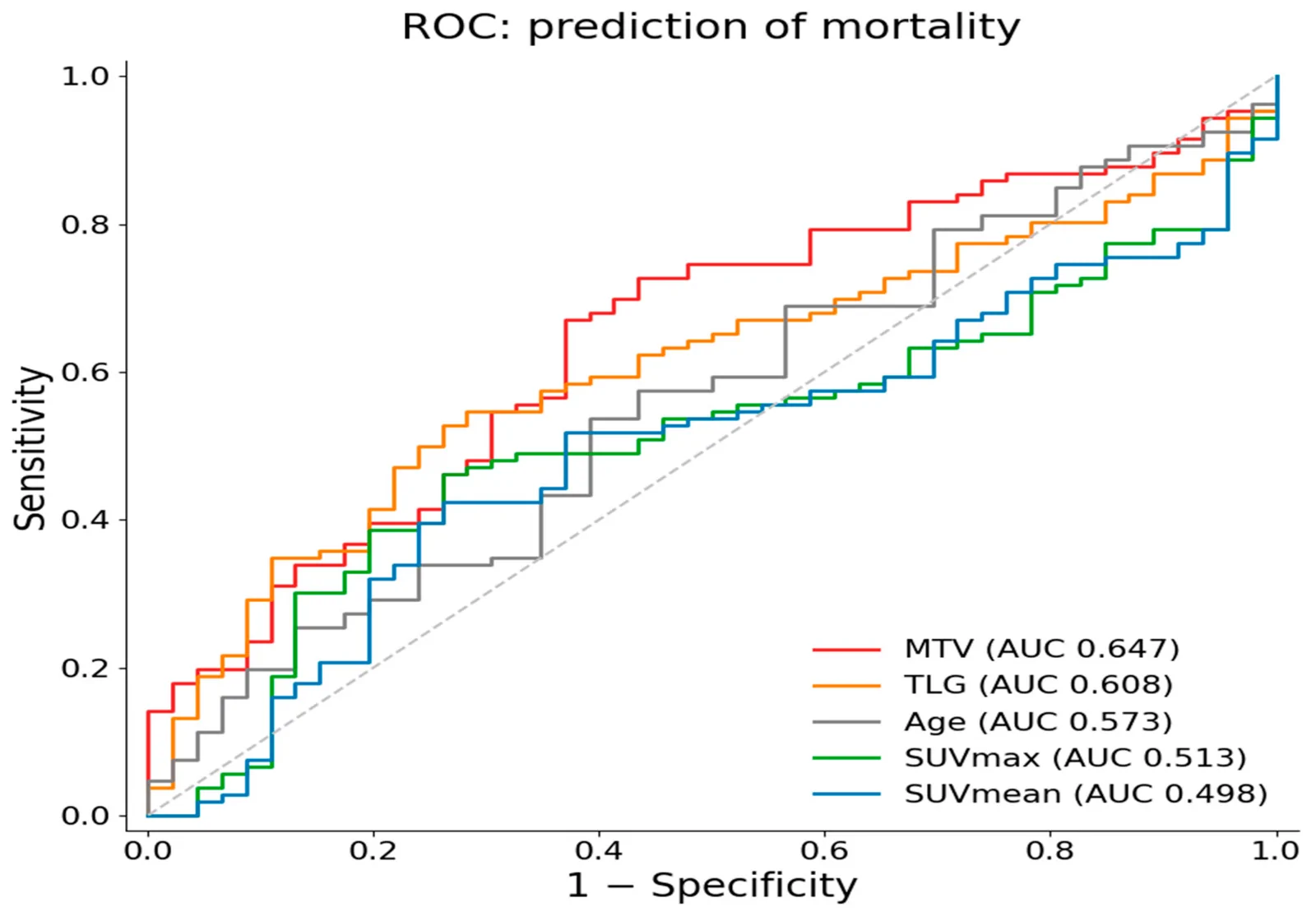
ROC curves for the prediction of mortality by pretreatment metabolic parameters and age.

Deceased patients had higher pretreatment MTV than survivors (median 15.06 vs. 9.20 cm^3^, *p* = 0.004) and higher TLG (131.76 vs. 74.26 g, *p* = 0.035), whereas the intensity-based indices did not differ between the two groups (SUVmax *p* = 0.81; SUVmean *p* = 0.97) (Table 2). In ROC analysis for mortality, MTV showed the highest discrimination (AUC 0.647), followed by TLG (0.608), whereas SUVmax (0.513), SUVmean (0.498), and age (0.573; deceased patients were older, median 66.0 vs. 61.5 years, *p* = 0.155; Table 2) were non-discriminative (Figure 4). In time-dependent ROC analysis accounting for censoring, the discrimination of MTV was highest for early mortality (AUC 0.731 at 12 months and 0.692 at 24 months) and attenuated at later horizons (0.578 at 60 months), with a similar pattern for TLG (0.739, 0.681, and 0.568, respectively); the ROC-derived cut-offs should therefore be regarded as exploratory.

**Figure 5:**
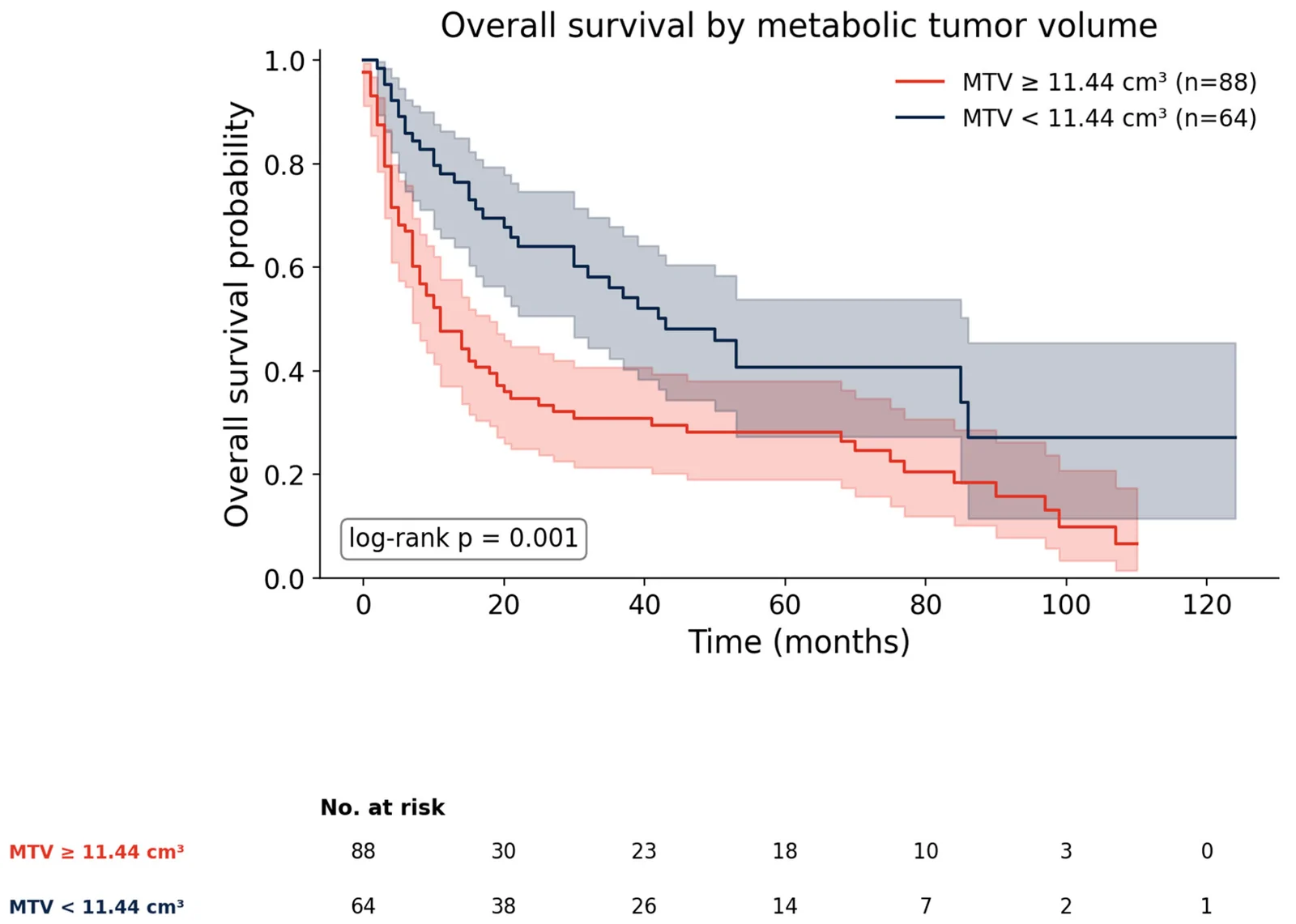
Overall survival stratified by metabolic tumor volume above versus below the ROC-derived cut-off. Numbers at risk are shown below the *x*-axis.

When dichotomized at their ROC-derived cut-offs, the parameters separated survival curves: high MTV (≥11.44 cm^3^) was associated with markedly shorter median OS (11.0 vs. 43.0 months; log-rank *p* = 0.001; Figure 5), as was high TLG (≥128.01 g; 9.0 vs. 43.0 months, *p* < 0.001). High SUVmax (≥16.85) also separated the curves (11.0 vs. 32.0 months, *p* = 0.014), although SUVmax was not associated with survival when analyzed as a continuous variable (HR 1.012 per unit, *p* = 0.39); all cut-offs are exploratory.

### 3.5. Independent Prognostic Factors (Cox Regression)

On univariable Cox analysis, distant metastasis (HR 3.11 (1.97–4.91), *p* < 0.001), nodal positivity (HR 2.01 (1.34–3.01), *p* < 0.001), male sex (HR 1.70 (1.15–2.51), *p* = 0.008), MTV (per cm^3^; HR 1.023, *p* < 0.001), and TLG (per g; HR 1.002, *p* < 0.001; equivalently, HR 1.18 (95% CI 1.10–1.26) per 100 g increment) were associated with OS, whereas SUVmax, SUVmean, age, and tumor location were not (Table 3). In the multivariable model (all 152 patients, 106 events; Harrell’s C 0.687), distant metastasis (HR 2.46 (1.53–3.98), *p* < 0.001) and high MTV (HR 1.79 (1.18–2.71), *p* = 0.006) remained independent predictors of death; nodal positivity and male sex showed non-significant trends. AJCC stage III–IV was likewise significant in univariable analysis (HR 2.15, 95% CI 1.42–3.25, *p* < 0.001) but was not included in the multivariable model owing to collinearity with the nodal and metastatic descriptors. In a complementary multivariable model in which AJCC stage group (III–IV vs. II) replaced the nodal and metastatic descriptors, both advanced stage (HR 1.80, 95% CI 1.17–2.76, *p* = 0.008) and high MTV (HR 1.81, 95% CI 1.20–2.73, *p* = 0.005) remained independently associated with OS (Harrell’s C 0.669); considered on their own, Harrell’s C was 0.600 for stage alone, 0.604 for dichotomized MTV alone, and 0.648 for the two combined. In bootstrap internal validation of the primary multivariable model, with re-derivation of the MTV cut-off in each of 1000 resamples, the optimism was small (ΔC = 0.021), yielding an optimism-corrected Harrell’s C of 0.666. Adding MTV to the stage-based model significantly improved model fit (likelihood-ratio χ^2^ = 8.21, *p* = 0.004). Formal assessment of multicollinearity confirmed that AJCC stage group III–IV was almost perfectly determined by the PET-derived nodal and metastatic descriptors (identical classification in all 152 patients; φ = 1.00; every stage-II patient was N0/M0, and every stage III–IV patient was N+ and/or M1 (the one apparent exception in an earlier data extract was traced to a stage-assignment transcription error, corrected upon re-verification against the source chart)), precluding their joint inclusion in a single model. In a separate multivariable model with the same clinical covariates, high TLG (≥128.01 g) was likewise independently associated with OS (HR 2.21, 95% CI 1.48–3.29, *p* < 0.001; all 152 patients), and this model achieved a Harrell’s C of 0.715, comparable to or higher than the primary MTV-based model (C = 0.687). To determine whether the prognostic value of MTV depended on the data-derived cut-off, we also fitted the primary multivariable model with MTV entered as a continuous variable: MTV remained independently associated with OS (HR 1.02 per cm^3^, 95% CI 1.01–1.03, *p* < 0.001; C = 0.705), confirming that the finding is not an artifact of dichotomization. Histological subtype was not associated with OS in univariable analysis (adenocarcinoma vs. SCC: HR 1.13, 95% CI 0.77–1.66, *p* = 0.54) and was therefore not carried forward. The proportional hazards assumption was not violated for any covariate (Schoenfeld *p* ≥ 0.06 for all); nonetheless, the time-dependent ROC results indicate that the discrimination of MTV is greatest early in follow-up, and the reported hazard ratios should be interpreted as time-averaged effects.

## 4. Discussion

In this single-center cohort of 152 treatment-naïve patients with esophageal cancer, with long-term follow-up, we evaluated baseline [^18^F]FDG PET/CT-derived metabolic parameters alongside clinicopathological variables to determine their prognostic significance for overall survival (OS). Metabolic tumor volume (MTV) and distant metastasis emerged as independent predictors of survival, whereas conventional intensity-based metrics such as SUVmax were not associated with survival. In a complementary model, high MTV retained independent prognostic value alongside AJCC stage group, suggesting that primary-tumor metabolic burden may refine rather than simply duplicate anatomical staging; because AJCC stage group is itself derived largely from nodal and metastatic status, and the PET-derived nodal/metastatic descriptors used here were near-collinear with stage (Section 2.1), this incremental-value claim should be regarded as provisional rather than a demonstration of information fully independent of staging. These findings reinforce the growing evidence that volumetric PET biomarkers convey prognostic information beyond anatomical staging alone [10,21,22].

The cohort was histologically mixed, comprising squamous cell carcinoma (SCC, 56.6%) and adenocarcinoma (43.4%); this balance is more representative of Western and transitional epidemiological settings than the squamous cell-dominant series that prevail in the East Asian literature [1,2]. Pretreatment metabolic parameters did not differ significantly between subtypes, and histological subtype was not associated with overall survival in univariable analysis (adenocarcinoma vs. SCC: HR 1.13, 95% CI 0.77–1.66, *p* = 0.54). Together, these observations suggest that quantitative assessment of tumor burden is more relevant to outcome than histological classification per se.

The AJCC eighth edition TNM system remains the cornerstone of prognostic assessment in esophageal cancer [3]. However, the variability in survival we observed within individual anatomical stages indicates that TNM staging alone does not fully capture tumor biology and highlights the complementary value of functional imaging biomarkers [4,7]. Integrated [^18^F]FDG PET/CT provides a combined anatomical and metabolic characterization of the primary tumor that anatomical staging alone cannot provide.

Although SUVmax is the most widely used PET metric, it reflects only the FDG-avid voxel with the highest SUV and does not represent total tumor burden [7,8,9]. In our cohort, SUVmax was not associated with survival as a continuous variable and discriminated poorly between survivors and non-survivors (AUC 0.513), suggesting that SUVmax reflects the intensity of FDG uptake in the most active focus rather than the extent of tumor present. In contrast, MTV and TLG quantify the entire metabolically active primary tumor, thereby providing a more biologically representative measure of primary-tumor metabolic burden [8,9,10]. Numerous individual series and recent meta-analyses have shown that elevated MTV and TLG predict poorer overall survival [6,10,15,16,17,21,22]. Consistent with these reports, MTV above its ROC-derived cut-off remained independently associated with an approximately 1.8-fold increase in mortality risk after adjustment for clinical covariates, including distant metastasis [14,15,16,17]. Although MTV independently predicted overall survival, its discriminatory performance as a single marker was only moderate (AUC = 0.647), and this deserves emphasis: taken alone, MTV misclassifies a substantial proportion of patients and should not be used as a stand-alone prognostic test; PET-derived metabolic tumor burden should complement rather than replace established clinicopathological prognostic factors. Accordingly, MTV is best interpreted as an adjunctive biomarker that refines risk stratification when integrated with conventional staging systems rather than as a stand-alone prognostic tool. Because TLG is mathematically derived from MTV and SUVmean, the two volumetric indices were evaluated in separate multivariable models rather than jointly; each remained independently prognostic with the same clinical covariates, consistent with their strong intercorrelation [10,16,17,21]. Discrimination of the two resulting models was comparable and, if anything, favored TLG: the TLG-based multivariable model achieved a Harrell’s C of 0.715, versus 0.687 for the primary MTV-based model. The present data therefore do not support ranking MTV above TLG as the better-performing PET parameter; both volumetric indices carried similar independent prognostic value, and either may be preferred depending on which is more readily available in a given workflow.

Several biological and technical considerations may explain why MTV outperforms SUVmax as a prognostic marker. SUVmax is derived from a single voxel and therefore captures only the peak glycolytic intensity of the most metabolically active tumor focus; it is inherently sensitive to image noise, reconstruction parameters, and partial-volume effects, and it conveys no information about how much metabolically active tumor is present. By contrast, MTV integrates FDG uptake across the entire lesion and thus approximates the total mass of viable, glycolytically active malignant cells within the primary tumor. A larger metabolically active volume reflects a greater proliferating cell burden and, plausibly, greater clonal diversity, a larger hypoxic and treatment-resistant fraction, and a higher probability of occult micrometastatic dissemination at diagnosis—all features that translate into poorer locoregional control and shorter survival. Clinically, this distinction matters: a small tumor with intense focal uptake (high SUVmax, low MTV) may still be highly curable, whereas a bulky lesion with only moderate uptake per voxel (lower SUVmax, high MTV) carries a substantially larger disease burden. Metabolic volume is therefore biologically closer to the concept of tumor burden that underlies anatomical staging, which may explain why MTV, but not SUVmax, retained independent prognostic significance in our multivariable model.

The prognostic utility of volumetric PET parameters has now been demonstrated across definitive chemoradiotherapy, trimodality therapy, and unresectable or metastatic settings [11,12,13,14], supporting further evaluation of volumetric PET parameters as adjunctive biomarkers in pretreatment risk assessment rather than as stand-alone prognostic tools.

All patients were managed under a standardized institutional chemoradiotherapy protocol, which limits variability attributable to differences in treatment regimen. Nevertheless, whether an individual patient ultimately proceeded to surgery or was managed with definitive or palliative intent, was determined by baseline stage, tumor burden, response to neoadjuvant therapy, and performance status. Treatment received is therefore partly a consequence of the same baseline characteristics whose prognostic value we evaluated, and adjusting for it in a baseline prognostic model would risk over-adjustment for a mediating variable; for this reason, treatment was not entered into the multivariable analysis. Residual confounding by treatment intent nonetheless cannot be excluded, and the prognostic value of MTV should be interpreted as applying to patients managed within such a protocol-based, multidisciplinary treatment pathway rather than to any single, uniformly treated therapeutic setting.

A principal strength of this study is its long-term follow-up in a contemporary cohort: patients were diagnosed between 2015 and 2025, with a median follow-up of 70.0 months and a maximum of 124 months—an observation window that exceeds that of most published PET prognostic series [13,18]. A further distinctive feature is that this Turkish tertiary-center cohort comprised both SCC and adenocarcinoma, complementing the predominantly East Asian, squamous cell series that dominate the existing literature. Notably, despite the cohort including both major histological subtypes in near-balanced proportions, histological subtype was not prognostic in univariable analysis. The principal contribution of the present study is independent confirmation of volumetric PET biomarkers in a Turkish, histologically mixed cohort with long-term follow-up, rather than methodological novelty.

Limitations include the retrospective single-center design and heterogeneity in treatment intent (surgical, definitive, or palliative management within the institutional protocol). The inclusion period was long (May 2015 to August 2025), during which staging procedures, systemic therapies, and radiotherapy techniques evolved considerably—most notably the introduction of immune checkpoint inhibitors into the management of advanced esophageal cancer—so era-related differences in diagnosis and treatment may have influenced survival outcomes. Two PET/CT systems were used over the study period without formal cross-calibration, which may have introduced variability into the quantitative parameters. The ROC-derived MTV cut-off was derived and applied within the same dataset; although bootstrap internal validation indicated only modest optimism, external validation in independent cohorts is required before any threshold can be recommended for clinical use; the MTV cut-off, like all data-derived thresholds in this study, should be regarded as exploratory. Interobserver reproducibility of the quantitative measurements was not formally assessed, and individual-level treatment intent could not be reliably reconstructed for the full cohort, so treatment-related confounding cannot be fully excluded. Because primary-tumor T category was not uniformly retrievable, the full TNM triad was not available, and residual confounding by primary-tumor extent cannot be excluded. Quantitative values were re-verified against the source records before analysis, so the final dataset is complete and no patient was excluded for data-quality reasons, but the residual risk of transcription error inherent to any retrospective database cannot be entirely excluded. Although follow-up was long, relatively few patients remained at risk beyond seven years, so the longest-horizon (≥9–10-year) estimates are based on small numbers. Strengths include standardized PET acquisition, comprehensive quantitative PET analysis, and long-term follow-up.

In conclusion, in this single-center cohort with long-term follow-up (median 70 months; maximum 124 months), baseline MTV was an independent predictor of overall survival alongside distant metastasis and retained prognostic value when modeled together with AJCC stage. Given the moderate discriminative performance of MTV as a single marker, these findings support its use as an adjunctive biomarker that refines, rather than replaces, established staging in pretreatment risk stratification. Prospective multicenter studies with external validation of standardized MTV thresholds are warranted to determine their integration into future prognostic models and clinical decision-making.

## Figures and Tables

**Table 1:** Baseline characteristics of the cohort. IQR, interquartile range; MTV, metabolic tumor volume; TLG, total lesion glycolysis.

Characteristic	Value (N = 152)
Sex—male/female, n (%)	80 (52.6)/72 (47.4)
Age, years—median (range)	63.0 (21–89)
Age ≥ 65—n (%)	73 (48.0)
Tumor location—upper, n (%)	21 (13.8)
middle, n (%)	55 (36.2)
lower, n (%)	76 (50.0)
Histology—SCC/adenocarcinoma, n (%)	86 (56.6)/66 (43.4)
AJCC stage—II, n (%)	62 (40.8)
III, n (%)	65 (42.8)
IV, n (%)	25 (16.4)
Nodal status (PET)—N0/N+, n (%)	65 (42.8)/87 (57.2)
Distant metastasis (PET)—M0/M1, n (%)	127 (83.6)/25 (16.4)
SUVmax—median (IQR)	13.99 (10.04–20.46)
SUVmean—median (IQR)	8.68 (5.72–12.16)
MTV, cm^3^—median (IQR)	12.83 (6.85–25.83)
TLG, g—median (IQR)	99.70 (43.40–202.20)
Deceased at data-cut—n (%)	106 (69.7)
Median follow-up, months	70.0

**Table 2:** Metabolic parameters and age by vital status, with ROC discrimination for mortality. MWU, Mann–Whitney U test; AUC, area under the ROC curve; cut-off by the Youden index.

Parameter	Alive, Median	Deceased, Median	*p* (MWU)	AUC	Cut-Off
SUVmax	13.56	14.52	0.808	0.513	16.85
SUVmean	8.36	8.96	0.968	0.498	10.41
MTV, cm^3^	9.20	15.06	0.004	0.647	11.44
TLG, g	74.26	131.76	0.035	0.608	128.01
Age, years	61.5	66.0	0.155	0.573	64

**Table 3:** Univariable and multivariable Cox proportional hazards analysis for overall survival. HR, hazard ratio; CI, confidence interval. Univariable hazard ratios are expressed per unit for continuous variables (age per year; MTV per cm^3^; TLG per g; SUVmax and SUVmean per unit). In the multivariable models, age and MTV were entered as dichotomized variables (age ≥ 65 years; MTV ≥ 11.44 cm^3^ at the ROC-derived cut-off), whereas sex, nodal status, and distant metastasis were categorical. Dashes indicate variables not entered in the multivariable model. The “Multivariable” column shown here is the primary, prespecified model (nodal status, distant metastasis, sex, age, MTV; Harrell’s C = 0.687). Two further models, not tabulated, used the same clinical covariates: one substituting AJCC stage group for the nodal/metastatic descriptors (Harrell’s C = 0.669) and one substituting high TLG (≥128.01 g) for MTV (HR 2.21, 95% CI 1.48–3.29, *p* < 0.001; Harrell’s C = 0.715); results of both are reported in Section 3.5.

Variable	Univariable HR (95% CI)	*p*	Multivariable
Age (univariable per year; multivariable ≥ 65 years)	1.01 (0.99–1.03)	0.251	1.04 (0.70–1.53) (*p* = 0.852)
Male sex	1.70 (1.15–2.51)	0.008	1.43 (0.96–2.13) (*p* = 0.083)
Nodal N+ (vs. N0)	2.01 (1.34–3.01)	<0.001	1.45 (0.94–2.24) (*p* = 0.091)
Distant metastasis M1	3.11 (1.97–4.91)	<0.001	2.46 (1.53–3.98) (*p* < 0.001)
MTV (univariable per cm^3^; multivariable ≥ 11.44 cm^3^)	1.023 (1.014–1.033)	<0.001	1.79 (1.18–2.71) (*p* = 0.006)
TLG (per g)	1.002 (1.001–1.002)	<0.001	—
SUVmax (per unit)	1.012 (0.985–1.039)	0.391	—
SUVmean (per unit)	1.014 (0.973–1.056)	0.514	—

## Data Availability

The raw data supporting the conclusions of this article will be made available by the authors on request.

## Supplementary Materials

The following supporting information can be downloaded at: https://www.mdpi.com/article/10.3390/diagnostics16172757/s1. Figure S1: Patient flow diagram.
